# Structural Characterization, In Vitro Digestion Property, and Biological Activity of Sweet Corn Cob Polysaccharide Iron (III) Complexes

**DOI:** 10.3390/molecules28072961

**Published:** 2023-03-26

**Authors:** Weiye Xiu, Xin Wang, Shiyou Yu, Zhiguo Na, Chenchen Li, Mengyuan Yang, Yongqiang Ma

**Affiliations:** Key Laboratory of Cereals and Comprehensive Processing of Cereal Resources, College of Food Engineering, Harbin University of Commerce, Harbin 150028, China

**Keywords:** sweet corn cob polysaccharide-iron (III) complexes, structural characterization, in vitro digestion property, in vitro antioxidant activity, in vitro hypoglycemic activity

## Abstract

This study aimed to enhance the utilization value of sweet corn cob, an agricultural cereal byproduct. Sweet corn cob polysaccharide-ron (III) complexes were prepared at four different temperatures (40 °C, 50 °C, 60 °C, and 70 °C). It was demonstrated that the complexes prepared at different temperatures were successfully bound to iron (III), and there was no significant difference in chemical composition; and SCCP-Fe-C demonstrated the highest iron content. The structural characterization suggested that sweet corn cob polysaccharide (SCCP) formed stable β-FeOOH iron nuclei with −OH and −OOH. All the four complexes’ thermal stability was enhanced, especially in SCCP-Fe-C. In vitro iron (III) release experiments revealed that all four complexes were rapidly released and acted as iron (III) supplements. Moreover, in vitro antioxidant, α-glucosidase, and α-amylase inhibition studies revealed that the biological activities of all four complexes were enhanced compared with those of SCCP. SCCP-Fe-B and SCCP-Fe-C exhibited the highest in vitro antioxidant, α-glucosidase, and α-amylase inhibition abilities. This study will suggest using sweet corn cobs, a natural agricultural cereal byproduct, in functional foods. Furthermore, we proposed that the complexes prepared from agricultural byproducts can be used as a potential iron supplement.

## 1. Introduction

Iron is essential for maintaining regular body activity and is abundant in the blood and liver [1]. Iron also serves a hematopoietic function in the human body. It is the material used for synthesizing hemoglobin which plays a role in transporting oxygen and nutrients. It is directly involved in oxygen storage and transport within the body. In addition, iron synthesizes hemoproteins, cytochromes, and various enzymes, enabling the smooth metabolism of the body [2,3]. In addition, it is necessary to produce or remove free radicals from the body. Iron deficiency has also been linked to oxidative stress in the body, which can lead to decreased immunity and disorders of glucose metabolism [4,5].

With advancements in research and development, many people have opted for supplementary preparations for iron supplements. Therefore, third-generation iron supplements are currently available. First-generation iron supplement preparations primarily refer to inorganic iron salts, such as ferrous sulfate, ferrous chloride, and ferrous carbonate. These supplement preparations are inexpensive but have poor drug stability, low iron bioavailability, and cause gastrointestinal irritation [6]. Therefore, second-generation iron supplements have gradually entered the market to improve iron bioavailability. These iron supplements are mostly solubilized small-molecule organic acids and iron salt chelates, such as ferrous gluconate and ferrous sodium iron. These iron supplements have improved iron bioavailability compared with first-generation inorganic iron supplements, but the taste could be better, which is not conducive to their application in food. Polysaccharide iron complexes belong to the third generation of iron supplements and are composed of trivalent iron and polysaccharide complexes. They have both ideal bioactivity and high bioavailability [7,8].

Polysaccharides are carbohydrates with complex molecular structures found in nature. They are structurally complex sugars formed by the condensation and water loss of more than 10 monoglycoside units, and they have a variety of biological activities. It has been found that polysaccharides can be extracted from agricultural byproducts [9]. In recent years, sweet corn has maintained an upward trend in agricultural product consumption, and its use has increased [10]. As a result, the hoarding of sweet corn byproducts such as sweet corn cobs, sweet corn husks, and sweet corn whiskers has increased. Sweet corn cobs are cobs after the seeds have been removed and threshed. The exploitation of sweet corn cob resources must be improved, as most are left unused, burned, or used as agricultural fertilizers [11].

We previously extracted sweet corn cob polysaccharides and investigated their functional properties [12]. Some researchers found that polysaccharide-iron (III) complexes would enhance bioactivity and could be used as a potential iron supplement. Zhang et al. prepared a *Poria cocos* polysaccharide-iron (III) complex. They found that it showed a better ability to scavenge free radicals and better antioxidant activity than Poria cocos polysaccharides and they suggested the form of β-FeOOH might affect the stability of the complex [13]. Furthermore, another polysaccharide-iron (III) complex prepared with a polysaccharide from pacific abalone showed that iron (III) was combined with polysaccharide by the Fe-O bond. This polysaccharide-iron (III) complex was also determined to have excellent iron-releasing ability and has great potential to be applied as a good iron supplement, which might related to its structure [14].

To enhance the bioavailability of sweet corn cob polysaccharides and develop an iron supplement based on polysaccharides, we hypothesized that the complex has higher iron and more potent antioxidant and hypoglycemic properties than polysaccharides alone. We used an agricultural byproduct, sweet corn cob, and assumed that the polysaccharides obtained from it could form polysaccharide-iron (III) complexes. Sweet corn cob polysaccharide-iron (III) complexes were prepared at four different temperatures, and their structural differences were analyzed. In addition, the in vitro release of iron (III) and the biological activity of four sweet corn cob polysaccharide-iron (III) complexes were investigated. This study will improve the utilization of sweet corn cobs in developing iron supplements and antioxidant and hypoglycemic functional foods.

## 2. Results and Discussion

### 2.1. Determination of Chemical and Monosaccharide Compositions

Table 1 lists the chemical and monosaccharide compositions of the samples at different temperatures. The total carbohydrate, protein, uronic acid, and reducing sugar contents of the four polysaccharide-iron (III) complexes did not differ significantly (*p* > 0.05). The monosaccharide composition results reveal that the polysaccharide-iron (III) complexes were primarily composed of glucose, nearly identical to the composition of SCCP monosaccharides. Interestingly, the contents of mannose, glucose, and fucose in the four complexes decreased compared to those in SCCP, whereas galactose and arabinose increased slightly. This could be related to the alkaline environment created during preparation. In short, the elemental chemical composition of SCCP does not change significantly during the preparation of the four polysaccharide-iron (III) complexes [15]. Therefore, we speculate that the introduction of iron (III) may not cause significant structural changes in the polysaccharide.

The iron (III) content of the four polysaccharide-iron (III) complexes prepared at different temperatures was 12.75 ± 1.26%, 15.67 ± 1.48%, 21.09 ± 2.29%, and 14.82 ± 1.31%, respectively. It showed an increasing trend and then a decreasing trend. This could be influenced by the increased polysaccharide motion rates with increasing temperature and the possibility of increasing collisional binding. However, the content decreased significantly when the temperature was excessively high, owing to the high molecular motion rate [16,17].

### 2.2. SEM-EDS Analysis

**Figure 1:**
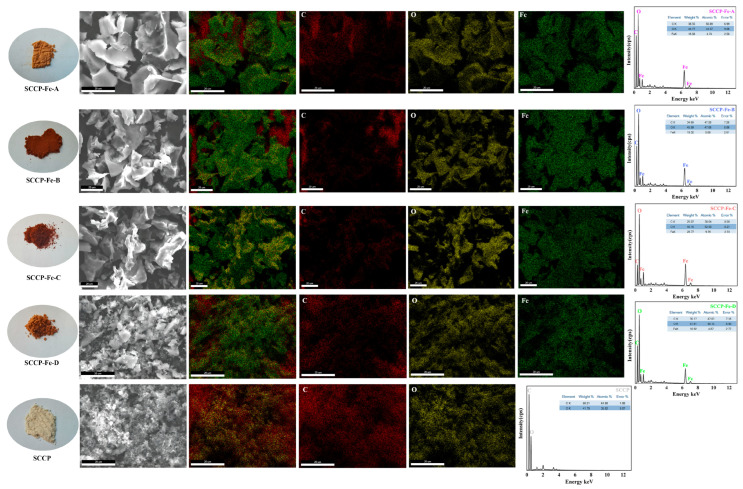
SEM-EDS analysis of four sweet corn cob polysaccharide-iron (III) complexes (physical images, microscopic morphology and scanned images of C, O, and Fe elements).

The result shows that SCCP was a white powder. Sweet corn cob polysaccharide-iron (III) complexes appeared as brown powders after complexation with iron (III) (Figure 1). This indicates that the introduction of iron (III) brought about changes in the appearance of the polysaccharide. To further determine the microscopic morphology of the complexes and the presence of iron (III) in the complexes, an SEM-EDS analysis method was used. A SEM-EDS analysis was used to examine the elements present and their distribution in the polysaccharide-iron (III) complexes. The surface morphologies of the complexes and SCCP revealed irregular structures in the form of flakes or crumbs. The smoothness of the polysaccharide-iron (III) complexes was significantly improved compared with that of SCCP. This could be due to the introduction of iron connecting the adjacent sugar chains and the cross-linking of the molecular chains to form aggregates. It also indicates that the introduction of iron does not significantly affect the morphological structure of SCCP [18,19]. To demonstrate the successful synthesis of the polysaccharide-iron (III) complexes, the elements were further scanned by X-ray energy dispersive spectrometry (EDS), and the percentage of each element was determined. The weight percentages of Fe in the four polysaccharide-iron (III) complexes were 16.68%, 19.32%, 28.77%, and 16.92%, with elemental percentages of 4.74%, 5.66%, 9.39%, and 4.87%, respectively. This indicates that iron (III) was bound to SCCP to form a complex, whereas its current form requires further structural characterization.

### 2.3. Ultraviolet (UV) Spectroscopy Analysis

**Figure 2:**
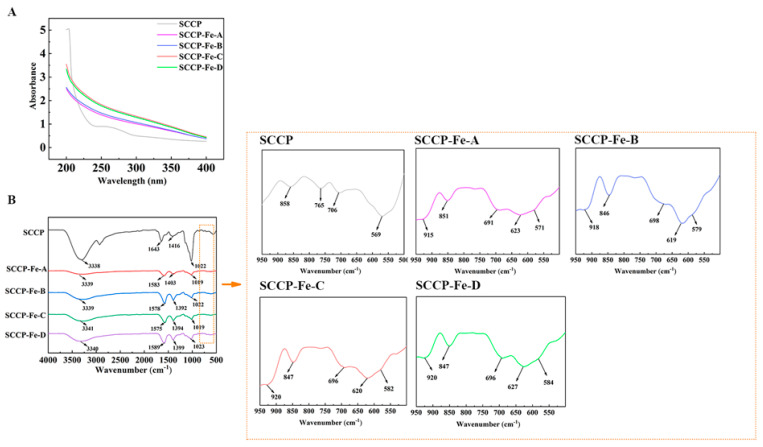
UV-vis (**A**) and FT-IR spectrum (**B**) of four sweet corn cob polysaccharide-iron (III) complexes.

The UV spectra of the sweet corn cob polysaccharide-iron (III) complexes are shown in Figure 2A. No significant peaks were detected at 260 or 280 nm, indicating that sweet corn cob polysaccharide-iron (III) complexes contain almost no proteins or nucleic acids. It was also found that the absorbance values were significantly higher in the 200–400 nm range than in the SCCP, which could be attributed to the Fe (III) interaction in sweet corn cob polysaccharide-iron (III) complexes compared with the SCCP group. A polysaccharide-iron (III) complex from *Glehniae Radix* yielded similar results [20]. Polysaccharide-iron (III) complexes could all be formed at temperatures ranging from 40–70 °C. To determine the presence of iron (III) in the polysaccharide-iron (III) complex form and the induced changes in the structure, FT-IR and XPS experiments were performed.

### 2.4. Fourier Transform Infrared (FT-IR) Spectroscopy Analysis

Figure 2B shows the FT-IR spectra of the sweet corn cob polysaccharide-iron (III) complexes. Compared with SCCP, the prominent absorption peaks did not change remarkably in the polysaccharide-iron (III) complexes prepared at four different temperatures. The absorption peak near 3340 cm^−1^ was mainly caused by the −OH stretching vibration, and the absorption peak near 1650 cm^−1^ was mainly due to the C=O asymmetric vibration of −COOH in the polysaccharide [21]. It also shows that the absorption peak around 1400 cm^−1^ reflects the symmetric vibration of C−O in −COOH. Interestingly, these two peaks indicate a significant shift in sweet corn cob polysaccharide-iron (III) complexes, which could be attributed to the introduction of iron (III), which influenced the structure of −COOH. The asymmetric stretching vibration of the pyranose ring in the polysaccharide appeared near 1020 cm^−1^, and the peak at 850 cm^−1^ was associated with the α-glycosidic bond in the pyranose ring [16,22]. In the fingerprint region from 900 to 600 cm^−1^, the absorption peak shape of the sweet corn cob polysaccharide-iron (III) complexes also changed compared to that of SCCP. The distinct absorption peaks at 900 cm^−1^ and 695 cm^−1^ were similar to those of β-hydroxy iron oxide (β-FeOOH) and presumably the formation of β-FeOOH iron nuclei. Similar results have been reported for a polysaccharide-iron (III) complex from *Flammulina velutipes* [14].

### 2.5. XPS Analysis

**Figure 3:**
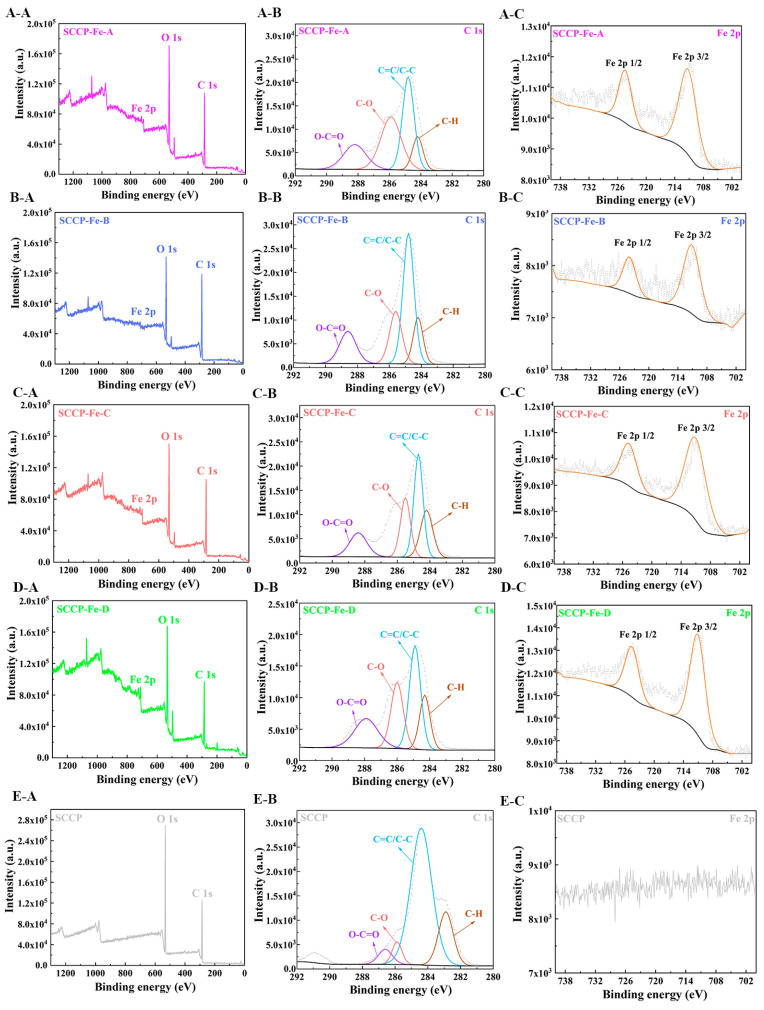
XPS analysis of four sweet corn cob polysaccharide-iron (III) complexes ((**A-A**) XPS spectra of the total elements; (**A-B**) C 1s; (**A-C**) Fe 2p, in SCCP-Fe-A; (**B-A**) XPS spectra of the total elements; (**B-B**) C 1s; (**B-C**) Fe 2p, in SCCP-Fe-B; (**C-A**) XPS spectra of the total elements; (**C-B**) C 1s; (**C-C**) Fe 2p, in SCCP-Fe-C; (**D-A**) XPS spectra of the total elements; (**D-B**) C 1s; (**D-C**) Fe 2p, in SCCP-Fe-D; (**E-A**) XPS spectra of the total elements; (**E-B**) C 1s, (**E-C**) Fe 2p, in SCCP).

The surface elements and forms present in the compounds were identified using X-ray photoelectron spectroscopy (XPS). According to the XPS full-scan spectra (Figure 3), all four polysaccharide-iron (III) complexes contained C, O, and Fe. Simultaneously, the characteristic peak of iron (III) was not detected in the SCCP spectra, indicating that iron (III) was successfully introduced into the sweet corn cob polysaccharide. The polysaccharides and forms of C and Fe present in the complexes were further characterized. In the C 1s spectrum, O−C=O, C−O, C=C/C−C, and C−H characteristic peaks were observed for SCCP and the complexes, indicating that the basic structure of the polysaccharide remained nearly unchanged. In addition, four polysaccharide-iron (III) complexes showed characteristic electron-binding energy peaks of Fe around 711 and 724 eV in the Fe 2p scan spectrum, indicating the introduction of iron [23]. This result corresponds with the FT-IR results. Under four different temperature conditions, iron (III) was structurally bound to polysaccharides. Moreover, we investigated the influence of the introduction of iron (III) on the three-stranded helix and the crystal structure of SCCP.

### 2.6. Congo Red Test

**Figure 4:**
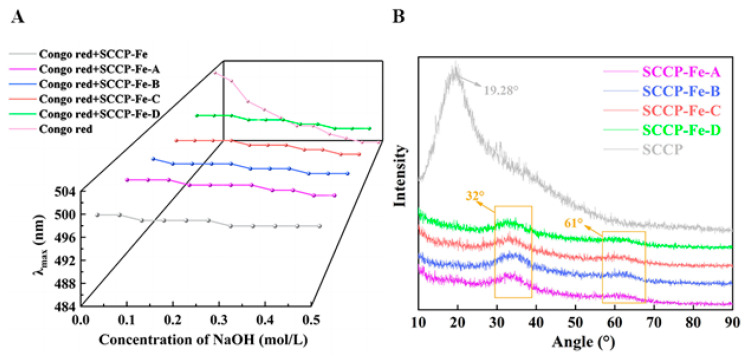
Congo red test (**A**) and XRD analysis (**B**) of four sweet corn cob polysaccharide-iron (III) complexes.

Congo red forms polysaccharides complexes with a three-stranded helical structure. The maximum absorption wavelengths of the complexes red-shifted under alkaline conditions as the NaOH content increased [24]. However, the maximum absorption wavelengths of the four sweet corn cob polysaccharide-iron (III) complexes and SCCP were not red-shifted, as shown in Figure 4A, indicating that the four complexes did not have a three-stranded helical structure. This also indicates that the introduction of iron (III) did not affect the three-stranded helical structure of SCCP. The triple helix structure of polysaccharides and their complexes have been linked to the monosaccharide composition. Heteropolysaccharides generally do not form triple helix structures. We also found four sweet corn cob polysaccharide-iron (III) complexes with a monosaccharide composition similar to SCCP. X-ray diffraction (XRD) experiments were performed to investigate their structural features further.

### 2.7. X-ray Diffraction Analysis

The crystal structures of the polysaccharides and their complexes were explored using XRD analysis. Figure 4B shows the XRD results for the polysaccharide-iron (III) complexes used in this study. In general, a broad diffraction peak indicated an amorphous structure. A broader peak in SCCP was found near 19.28°, indicating that SCCP has a non-crystalline amorphous structure. The complexes lacked a prominent sharp absorption peak compared with that of SCCP, and the peak at 19.28° was also significantly weakened. It reflected the fact that the introduction of iron (III) could affect the crystal structure of polysaccharide. In addition, two new characteristic peaks (32° and 61°) were observed in the polysaccharide-iron (III) complexes, indicating the presence of a β-FeOOH structure in the complexes [25]. This finding was consistent with the results obtained from the FT-IR analysis. Similar results were also obtained by Jing et al. for the preparation of a *Glehniae Radix* polysaccharide-iron complex [20]. The results showed that iron (III) was successfully introduced into SCCP and influenced the polysaccharide structure.

### 2.8. Thermal Gravimetric Analysis

**Figure 5:**
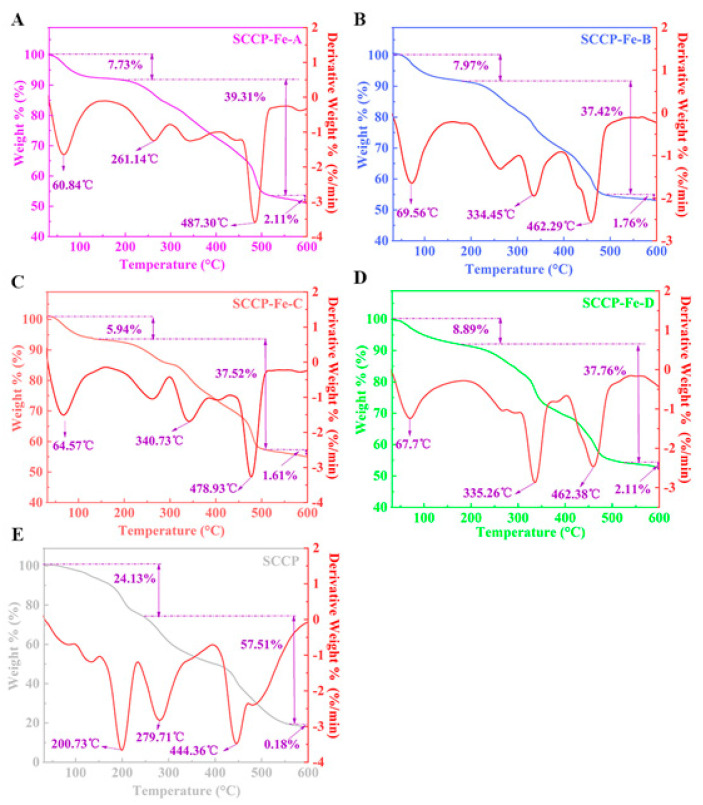
Thermal gravimetric analysis of four sweet corn cob polysaccharide-iron (III) complexes and SCCP ((**A**): SCCP-Fe-A; (**B**): SCCP-Fe-B; (**C**): SCCP-Fe-C; (**D**): SCCP-Fe-D; (**E**): SCCP).

According to the TG analysis shown in Figure 5, the four sweet corn cob polysaccharide-iron (III) complexes and SCCP had three primary heat-loss stages. The first loss stage ranged from 30 to 200 °C and was caused by the loss of free and bound water from the polysaccharides and their complexes [26]. We also found that four sweet corn cob polysaccharide-iron (III) complexes lost significantly less mass than SCCP in the first stage. Furthermore, the second loss stage occurs between 201 and 500 °C because of polysaccharide degradation. The mass loss was rapid during this stage. Chemical bond breakage and glycocyclic depolymerization occur in polysaccharides and their complexes, resulting in mass reduction due to the production of H_2_O, CO_2_, and other gases [27]. At this stage, the heat loss rates of the four sweet corn cob polysaccharide-iron (III) were 39.31%, 37.42%, 37.52%, and 37.76%, respectively, compared to 57.51% for SCCP. This could be due to the presence of Fe-O which improved thermal stability. The temperature of the third loss stage ranged from 501–600 °C. At this stage, the mass loss rate decreased, and the mass reduction was due to carbonization as the temperature increased.

At this stage, there was no significant difference in the mass loss between the four sweet corn cob polysaccharide-iron (III) complexes and SCCP. Overall, the mass loss rates of the four sweet corn cob polysaccharide-iron (III) complexes at 30–600 °C (48.75%, 47.15%, 45.07%, and 48.76%, respectively) were significantly lower than those of SCCP (81.82%). Thus, the sweet corn cob polysaccharide-iron (III) complex is more thermally stable. Moreover, introducing iron (III) binding to −OOH and −OH in polysaccharides leads to a more stable β-FeOOH iron core [28]. Therefore, it can significantly improve the stability of the complex compared to that of SCCP. We also found that there might be a positive correlation between the iron (III) content in the complexes and the β-FeOOH iron core structure, which can enhance the thermal stability of the complex [18]. Furthermore, this result was consistent with a soybean polysaccharide-iron (III) complex [29]. This result provides a foundation for further research into its in vitro release properties and bioactivity.

### 2.9. Determination of Simulated Digestion In Vitro

**Figure 6:**
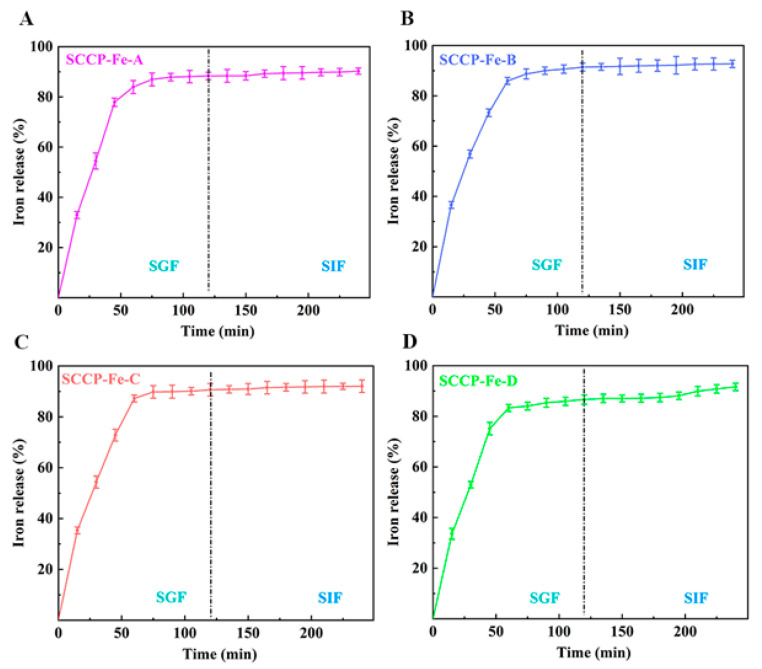
The iron (III) release in simulated gastric fluid (SGF) and simulated intestinal fluid (SIF) of four sweet corn cob polysaccharide-iron (III) complexes ((**A**): SCCP-Fe-A; (**B**): SCCP-Fe-B; (**C**): SCCP-Fe-C; (**D**): SCCP-Fe-D).

We performed experiments in SGF and SIF to determine the in vitro release rate of iron (III) from the prepared sweet corn cob polysaccharide-iron (III) complexes (Figure 6). The iron (III) release rates of all four complexes exceeded 80% during the first 60 min of SGF. Moreover, the total iron (III) release rates of the four complexes in simulated digestive juices were 90.27 ± 1.23%, 92.73 ± 1.47%, 92.05 ± 2.48%, and 91.62 ± 1.46%, respectively. This indicated that the release properties of all four complexes were satisfactory (*p* > 0.05). This also shows that the four different preparation temperatures did not affect the complexes’ iron (III) release.

Similar results were obtained by Liu et al. when measuring the in vitro iron (III) release of a polysaccharide-iron (III) complex in *Auricularia auricula*, which was consistent with our findings [30]. Furthermore, organic iron is rapidly released into gastric and intestinal fluids [31]. Therefore, the current study suggests that all four sweet corn cob polysaccharide-iron (III) complexes have good iron (III) release properties and can be used as potential iron supplements. Furthermore, the in vitro bioactivity was evaluated to investigate their utilization value.

### 2.10. Determination of Antioxidant Activity In Vitro

**Figure 7:**
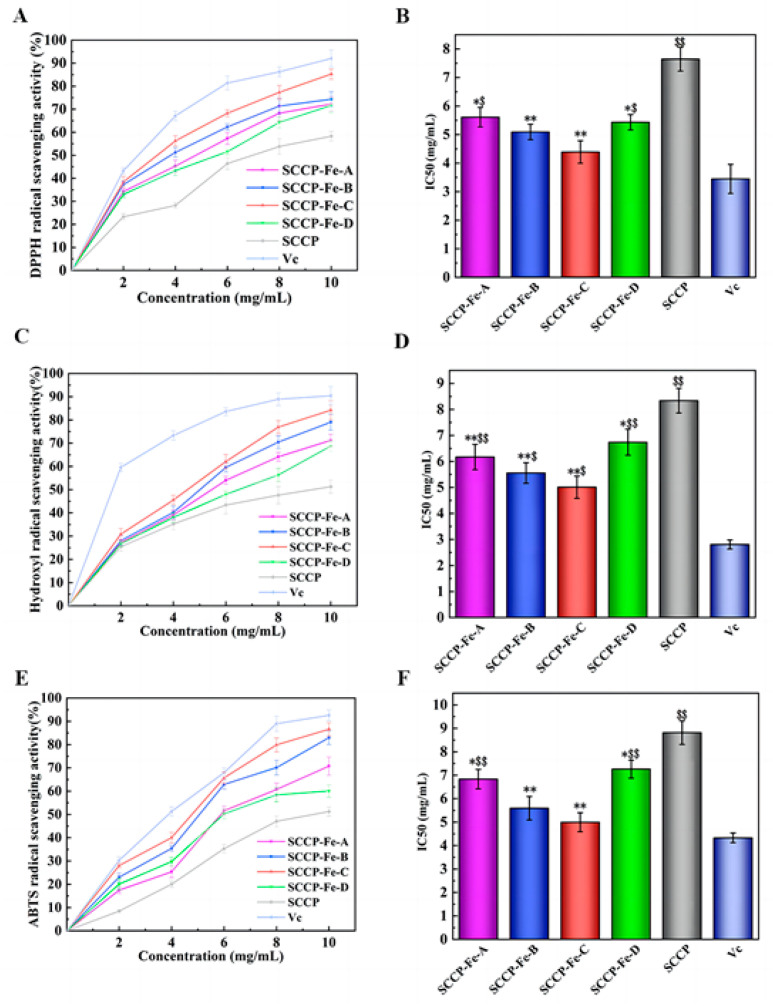
Antioxidant activity of four sweet corn cob polysaccharide-iron (III) complexes. ((**A**,**B**): DPPH radical-scavenging activity and its half-inhibition concentration (IC_50_); (**C**,**D**): hydroxyl radical scavenging activity and its IC_50_; (**E**,**F**): ABTS radical-scavenging activity and its IC_50_; *: *p* < 0.05 compared to SCCP; **: *p* < 0.01 compared to SCCP; $: *p* < 0.05 compared to Vc; $$: *p* < 0.01 compared to Vc.).

Figure 7A shows the DPPH radical scavenging rate of the sweet corn cob polysaccharide-iron (III) complex in vitro. The DPPH radical scavenging rate increased with increasing concentrations of the polysaccharide-iron (III) complex. For example, at a concentration of 10 mg/mL, the DPPH radical scavenging rates of the four sweet corn cob polysaccharide-iron (III) complexes were 72.29 ± 3.13%, 74.32 ± 3.29%, 85.25 ± 2.21%, and 71.62 ± 3.05%, respectively. Furthermore, we also calculated the IC_50_ value, since a smaller value indicates that fewer inhibitors are needed to achieve the free radical scavenging effect. It showed that the IC_50_ values were significantly lower for SCCP-Fe-B (5.092 ± 0.273 mg/mL) and SCCP-Fe-C (4.387 ± 0.395 mg/mL) than for SCCP (7.642 ± 0.416 mg/mL, *p* < 0.01), indicating that the polysaccharide-iron (III) complex had a remarkably lower DPPH radical scavenging capacity than SCCP (Figure 7B).

The ability to scavenge hydroxyl radicals indicated that the hydroxyl radical scavenging rate of the polysaccharide-iron (III) complexes increased with increasing concentration (Figure 7C). Moreover, at a concentration of 10 mg/mL, the scavenging rates of the four sweet corn cob polysaccharide-iron (III) complexes were 71.22 ± 2.66%, 79.04 ± 3.41%, 84.18 ± 4.06%, and 68.71 ± 2.25%, respectively. We also found that the IC_50_ values were significantly lower in SCCP-Fe-A (6.170 ± 0.485 mg/mL), SCCP-Fe-B (5.553 ± 0.392 mg/mL), and SCCP-Fe-C (5.01 ± 0.427 mg/mL) than in SCCP (8.338 ± 0.471 mg/mL), suggesting that the polysaccharide-iron (III) complex showed a significant increase in hydroxyl radical scavenging ability compared to SCCP (*p* < 0.01, Figure 7D).

The scavenging rate of ABTS radicals also increased with increasing concentration (Figure 7E). For example, the scavenging rates of ABTS radicals by four sweet corn cob polysaccharide-iron (III) complexes at a concentration of 10 mg/mL were 70.75 ± 3.93%, 82.95 ± 2.89%, 86.61 ± 2.82%, and 60.05 ± 2.63%, respectively. Moreover, the IC_50_ values were significantly lower (*p* < 0.01) than those of SCCP (8.815 ± 0.502 mg/mL), SCCP-Fe-B (5.593 ± 0.501 mg/mL), and SCCP-Fe-C (4.995 ± 0.407 mg/mL) as shown in Figure 7F. The results showed that the polysaccharide-iron (III) complex significantly enhanced ABTS’ radical scavenging ability.

All four sweet corn cob polysaccharide-iron (III) complexes tested positive for antioxidant activity. Dong et al. reported similar results [32]. Importantly, we also found that as the temperature of the complexes increased during the preparation process, the polysaccharide-iron (III) complexes showed an increasing and then decreasing trend in free radical scavenging ability. Therefore, this may be positively related to the iron (III) content of the complexes. Iron (III) is essential for free radical scavenging. Moreover, our previous study revealed that sweet corn cob polysaccharides have antioxidant activities. Therefore, introducing iron (III) synergizes with the free radical scavenging ability. In addition, it was also shown that polysaccharide coordination with iron (III) resulted in the formation of the β-FeOOH iron core, causing changes in the spatial structure of the polysaccharide and an increase in the number of free radical scavenging active sites [26].

Free radicals are chemically defined as groups containing unpaired electrons, which are produced by normal metabolic processes in the body. The mechanism of antioxidant activity of sweet corn cob polysaccharide-iron (III) complexes is twofold. Firstly, the −OH of the polysaccharide in the complex can be paired with the free radical lone pair electrons and thus has the activity of scavenging free radicals [33]. Moreover, it is also possible that the stable β-FeOOH iron core formed after the sweet corn cob polysaccharide-iron (III) complexes were more stable in spatial structure than SCCP, which is beneficial for the antioxidant capacity of polysaccharides [34]. These results were similar to those of some other studies. An *Auricularia auricula* polysaccharide-iron (III) complex was also found to have better antioxidant activity in vitro [30]. In addition, another polysaccharide-iron (III) complex from *Cordyceps militaris mycelia* also suggested that high activity in an antioxidant was present [35]. In summary, sweet corn cob polysaccharide-iron (III) complexes showed higher antioxidant activity than SCCP, with SCCP-Fe-C demonstrating the highest activity.

### 2.11. Determination of Hypoglycemic Activity In Vitro

Recently, the number of people with diabetes has increased annually. An effective way to relieve diabetes is to control and reduce blood sugar levels. The starch in food needs to be broken down in the intestine, and α-glucosidase and α-amylase play central roles in this. Polysaccharides can lower blood sugar levels by inhibiting α-glucosidase and α-amylase, which reduce glucose production in the body. Likewise, polysaccharides can reduce glucose production in the body by inhibiting α-glucosidase and α-amylase, thereby controlling the increase in blood glucose levels after a meal.

**Figure 8:**
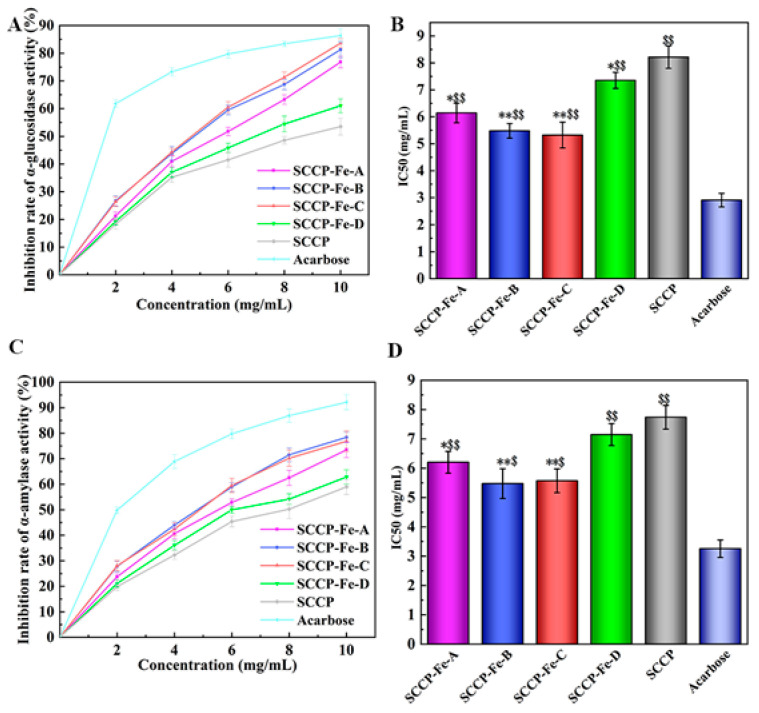
Hypoglycemic activity of four sweet corn cob polysaccharide-iron (III) complexes. ((**A**,**B**): The inhibition rate of α-glucosidase activity and its IC50. (**C**,**D**): The inhibition rate of α-amylase activity and its IC50. *: *p* < 0.05 compared to SCCP; **: *p* < 0.01 compared to SCCP; $: *p* < 0.05 compared to Acarbose; $$: *p* < 0.01 compared to Acarbose.).

The inhibition of α-glucosidase in vitro has also been demonstrated. Moreover, the results indicated that the four sweet corn cob polysaccharide-iron (III) complexes significantly increased α-glucosidase inhibition at the concentration of 10 mg/mL (Figure 8A). SCCP-Fe-B (81.29 ± 2.93%) and SCCP-Fe-C (83.64 ± 1.66%) showed the best inhibition rates. The IC_50_ values were 5.483 ± 0.272 mg/mL and 5.325 ± 0.479 mg/mL, respectively (Figure 8B). The result of α-amylase inhibition in vitro also showed that the inhibition rate of SCCP-Fe-B (78.39 ± 2.04%) and SCCP-Fe-C (76.84 ± 4.02%) was remarkably increased compared with SCCP at a concentration of 10 mg/mL (Figure 8C). The IC_50_ values were 5.475 ± 0.506 mg/mL and 5.571 ± 0.404 mg/mL, respectively (Figure 8D). These results indicated that the polysaccharide-iron (III) complex could enhance the ability of polysaccharides to inhibit α-glucosidase and α-amylase.

Polysaccharides contain multiple pathways in the inhibition of glycolytic enzyme activity. Firstly, polysaccharides may reduce the activity of the enzyme through direct inhibition, which may alter the conformation of the enzyme. In another way, polysaccharides may also compete for substrate binding, preventing the enzyme from binding to normal substrates or reducing its ability to bind. Simultaneously, polysaccharides may also bind to enzyme-substrate complexes. Naturally, polysaccharides may act in multiple ways simultaneously to achieve the inhibition of enzyme activity [36,37]. After binding with iron to form polysaccharide-iron complexes, this activity enhanced. It might be related to the increase in the iron (III) content of the complex at different preparation temperatures, which also verified our speculation that a more stable structure of the polysaccharide-iron (III) complex was favorable to its bioactivity [38]. The results indicate that SCCP-Fe-B and SCCP-Fe-C have better in vitro hypoglycemic activity, which provides context for their future development in functional foods.

## 3. Materials and Methods

### 3.1. Materials and Reagents

The sweet corn cobs (*Zea mays* L. saccharata Sturt) were obtained from Harbin HaoWei Agriculture Development, Ltd. Co. (Harbin, Heilongjiang, China). 2,2-Diphenyl-1-picryl-hydrazyl-hydrate (DPPH), 2,2′-azino-bis (3-ethylbenzothiazoline-6-sulfonic acid) (ABTS), α-amylase (50 U/mg), α-glucosidase (50 U/mg), acarbose, and 4-nitrophenol-α-D-furan glucoside (pNPG) were purchased from Macklin Inc. (Macklin, Shanghai, China). Standard monosaccharides (l-gulonic acid, d-mannuronic acid, d-mannose, d-ribose, l-rhamnose, d-glucosamine, d-glucuronic acid, d-galacturonic acid, d-glucose, d-galactosamine, d-galactose, d-xylose, d-arabinose, and d-fucose) were purchased from Sigma-Adrich Chemical Co. (St. Louis, MO, USA). All the other chemical reagents used were of an analytical grade.

### 3.2. Preparation of the Sweet Corn Cob Polysaccharide-Iron (III) Complex

**Figure 9:**
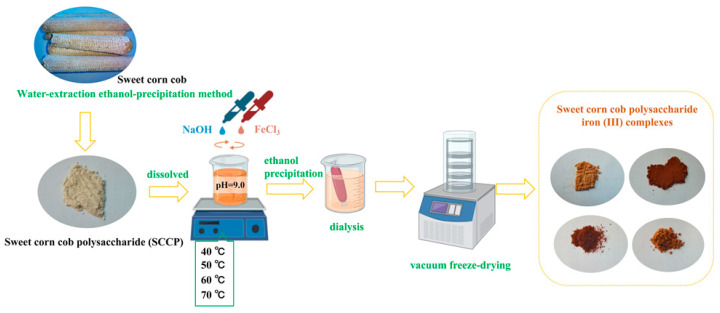
Schematic representation of the preparation steps for the sweet corn cob polysaccharide-iron (III) complexes.

The sweet corn cob polysaccharides were prepared using the method described by Wang et al. with appropriate modifications [39]. Briefly, the dried sweet corn cobs were crushed after defatting and extracted at 100 °C for 4 h. After decolorization, deproteinization, and alcoholic precipitation, the polysaccharides were extracted and named SCCP. The sweet corn cob polysaccharide-iron (III) complexes were prepared according to a previous report by Shi et al. [40]. At first, SCCP was mixed with three times the mass of SCCP in trisodium citrate, dissolved in distilled water, and FeCl_3_ (2 M) and NaOH (0.5 M) were added to the solution. The reaction pH was set to 9.0 until a brownish-red precipitate formed in the system, and the reaction temperatures were set to 40 °C, 50 °C, 60 °C, and 70 °C. The reaction mixture was continuously stirred for 50 min. The solution was centrifuged after cooling to room temperature, and the upper layer of brownish-red liquid was collected. Then, four times the volume of ethanol was added, and the mixture was left overnight. The precipitate was then centrifuged and freeze-dried. The complexes were then redissolved in distilled water, dialyzed for 48 h (2000 Da), and freeze-dried. The sweet corn cob polysaccharide-iron (III) complexes were obtained and named SCCP-Fe-A, SCCP-Fe-B, SCCP-Fe-C, and SCCP-Fe-D (Figure 9).

### 3.3. Characterization of the Sweet Corn Cob Polysaccharide-Iron (III) Complex

#### 3.3.1. Determination of the Chemical and Monosaccharide Compositions

The chemical composition of the sweet corn cob polysaccharide-iron (III) complexes was determined using previous studies reported by other researchers [41]. The iron (III) content was determined using the 1, 10-phenanthroline method, the sweet corn cob polysaccharide-iron (III) complexes (1 mg/mL, 0.1 mL) were added to a 1, 10-phenanthroline solution (2.5 mL) and ascorbic acid (0.1 g/mL, 2 mL), reacted at 37 °C for 10 min, and the absorbance values were measured at 510 nm. The total carbohydrate content was determined using the phenol-sulfuric method. The complexes (1 mg/mL, 2 mL) were added with concentrated sulfuric acid and phenol, and their absorbance value at 490 nm was measured. The protein content was analyzed with an adye-binding method with Coomassie Brilliant Blue G250 and detected at 595 nm. The uronic acid was detected using the sulfuric acid carbazole method; the complexes (1 mg/mL, 2 mL) were added sequentially with concentrated sulfuric acid and carbazole reagent, and the absorbance value at 530 nm was detected. The reducing sugar contents were detected with a 5-dinitro salicylic (DNS) method; the complexes (1 mg/mL, 0.5 mL) were added to the DNS reagent and then boiled for 5 min, and the absorbance was measured at 530 nm after cooling [42,43].

The monosaccharide composition of the complexes was determined by HPLC using a previously reported procedure with some modifications [44]. First, the sample (10 mg) was mixed with trifluoroacetic acid (TFA, 2 M, 5 mL) at 100 °C and hydrolyzed for 2 h. Then, 1 mL of the solution was removed, and an equal volume of methanol was added and evaporated with N_2_ to avoid the air. This procedure was repeated thrice to remove the residual TFA. Next, the hydrolysis solution was obtained after adding NaOH (0.3 M, 1 mL) to dissolve the residue. Then, the polysaccharide hydrolysate (0.4 mL) was mixed with 0.4 mL of 3-Methyl-1-phenyl-2-pyrazolin-5-one (PMP) methanol solution and incubated at 70 °C for 2 h. Finally, the aqueous phase was extracted with chloroform by adding hydrochloric acid (0.3 M) to adjust the pH to 6 and filtered through a membrane (0.45 µm). The sample was analyzed using a high-performance liquid chromatographic system (Agilent 1200, Agilent Technologies, Santa Clara, CA, USA) with a C18 column (4.6 mm × 250 mm, 5 µm; Agilent Technologies, USA) and UV detection at 250 nm, with a mobile phase of sodium phosphate buffer (pH = 6.7, 0.1 M, eluent A) and acetonitrile (eluent B). The gradient started with 14% eluent B, progressed to 17% in 9 min, 22% in 19 min, and 50% in 1 min, and was held for 2 min. Then, the column temperature was set to 30 °C, and the flow rate was set to 1 mL/min.

#### 3.3.2. SEM-EDS Analysis

An elemental analysis was performed using a scanning electron microscope (SEM) (SU8100, Hitachi Ltd., Tokyo, Japan) equipped with an EDAX EDS detector (EDAX Inc., Mahwah, NJ, USA). A small amount of the sweet corn cob polysaccharide-iron (III) complex powder (5 mg) was fixed on a sample plate with a double-sided conductive adhesive. The powder was gold-sputtered and characterized at 20.0 kV at different magnifications. Moreover, the distribution and content of iron have previously been analyzed [45,46].

#### 3.3.3. Ultraviolet (UV) Spectroscopy Analysis

Ultraviolet (UV) spectroscopy was performed using an ultraviolet spectrophotometer (TU-1900; Beijing Persee General Instrument Co., Ltd., Beijing, China). Four different polysaccharide-iron (III) complexes (20 mg) were dissolved in distilled water (2 mg/mL) and detected at 190–400 nm.

#### 3.3.4. Fourier Transform Infrared (FT-IR) Spectroscopy Analysis

A Fourier transform infrared (FT-IR) spectrometer (Perkin-Elmer 2000, Perkin Elmer Company, Waltham, MA, USA) was used to record the FT-IR spectroscopy analysis. Briefly, four different polysaccharide-iron (III) complexes (20 mg) were mixed, ground with KBr powder, and pressed into pellets. The scanning range was 4000–500 cm^−1^.

#### 3.3.5. XPS Analysis

X-ray photoelectron spectroscopy (XPS) was conducted using an XPS ESCALAB 250Xi instrument (Thermo Scientific, Waltham, MA, USA). A monochromatic Al Kα source (1486.8 eV, 15 kV, 10 mA) was used. The XPS analysis was performed to determine the bound form of iron in the polysaccharide-iron (III) complexes [47].

#### 3.3.6. Congo Red Test

Four polysaccharide-iron (III) complexes (2 mg/mL) were mixed with a Congo red solution (80 µmol/L, 2 mL) and NaOH (1 mol/L). Distilled water was added to achieve the final NaOH concentrations of 0, 0.05, 0.010, 0.15, 0.20, 0.25, 0.30, 0.35, and 0.40 mol/L. The mixture was reacted at 25 °C for 5 min, and the maximum absorption wavelength was recorded using UV full-spectrum scanning (Lambda 365, Perkin Elmer Instruments Co., Ltd., Waltham, MA, USA) in the wavelength range 400–600 nm [48].

#### 3.3.7. X-ray Diffraction Analysis

The crystallinity of the polysaccharide-iron (III) complexes was determined using an X-ray diffractometer (XRD, XRD-6000, Shimadzu Co., Tokyo, Japan). The test conditions were Cu-Kα1 (λ = 1.5406 nm) at a voltage of 40 kV and current of 40 mA, a scanning range of 5–90°, and a scanning speed of 5°/min [49].

#### 3.3.8. Thermal Stability Assay

A thermal stability analysis (TGA) of the polysaccharide-iron (III) complexes was performed using a thermogravimeter (STA 6000 Perkin Elmer Instruments GmbH, Rodgau, Germany). Briefly, the complex (5 mg) was placed in a sample pool, the temperature was set in the range of 30–600 °C, and the heating rate was set at 10 °C/min [50].

### 3.4. Determination of Simulated Digestion In Vitro

Simulated saliva fluid (SSF), simulated gastric fluid (SGF), and simulated intestinal fluid (SIF) was prepared as described by Zhu et al. [51]. For the preparation of SSF, α-amylase (2 mg/mL) was added to a salivary electrolyte (pH = 6.9), which consisted of NaCl (0.12 mg/mL), KCl (0.15 mg/mL), and CaCl_2_.2H_2_O (1 mg/mL). The SGF was composed of NaCl (2 mg/mL), CaCl_2_.2H_2_O (0.15 mg/mL), KCl (1 mg/mL), NaHCO_3_ (0.5 mg/mL), gastric lipase, and pepsin (0.15 mg/mL) and then adjusted to pH = 1.2 by HCl, and the SIF was composed of NaCl (5 mg/mL), KCl (0.65 mg/mL) CaCl_2_.2H_2_O (0.3 mg/mL), and trypsin (5 mg/mL) and adjusted to pH = 7.0 by NaOH (0.1 mol/L). Then, four polysaccharide-iron (III) complexes (10 mg/mL, 20 mL) were placed in dialysis bags. Subsequently, 50 mL of SGF was added, and the mixture was digested for 2 h. Then, 50 mL of SIF was added, and the mixture was incubated for another 2 h. The reaction was performed in a water bath at 37 °C with shaking at 100 rpm. Samples (5 mL) were taken at 15 min intervals and supplemented with the corresponding simulated digestion solution. The iron release was measured using the 1, 10-phenanthroline method.

### 3.5. Determination of Antioxidant Activity In Vitro

#### 3.5.1. 2,2-Diphenyl-1-picryl-hydrazyl (DPPH) Radical Scavenging Activity

Four polysaccharide-iron (III) complexes (0–10 mg/mL, 2 mL) were reacted with DPPH (0.2 mM, 2 mL dissolved in anhydrous ethanol) and protected from light for 0.5 h. The absorbance was recorded at 517 nm, and the scavenging activity was calculated using Equation (1). The concentration of free radicals at 50% scavenging (IC_50_) was also predicted. Equal concentrations of SCCP were prepared and measured in the same manner; the same concentration of ascorbic acid served as a positive control [52].

(1) $$\mathrm{DPPH}\;\mathrm{radical}\;\mathrm{scavenging}\;\mathrm{activity}(\%)=(1-\frac{A_{1}-A_{2}}{A_{0}})\times 100$$

where *A*_0_ is the absorbance without the sample, *A*_1_ is the absorbance of the polysaccharide-iron (III) complexes, and *A*_2_ is the absorbance without the DPPH-ethanol solution.

#### 3.5.2. Hydroxyl Radical Scavenging Activity

Four polysaccharide-iron (III) complexes (0–10 mg/mL, 2 mL) were mixed with ferrous sulfate (6 mmol/L, 2 mL), salicylic acid-ethanol (6 mmol/L, 2 mL), and hydrogen peroxide solutions (30%, 2 mL). The reaction was performed at 37 °C for 0.5 h, protected from light, and the absorbance was measured at 510 nm. The scavenging activity was calculated using Equation (2), and the IC_50_ value was predicted. Equal concentrations of SCCP were prepared and measured in the same manner; the same concentration of ascorbic acid served as a positive control [53].

(2) $$\mathrm{Hydroxyl}\;\mathrm{radical}\;\mathrm{scavenging}\;\mathrm{activity}\;(\%)=(1-\frac{A_{1}-A_{2}}{A_{0}})\times 100$$

where *A*_0_ is the absorbance without the sample, *A*_1_ is the absorbance of the polysaccharide-iron (III) complexes, and *A*_2_ is the absorbance without salicylic acid.

#### 3.5.3. 2,2′-Azino-bis (3-ethylbenzothiazoline-6-sulfonic Acid) (ABTS) Radical Scavenging Activity

An ABTS reagent was prepared according to a previously described method [54]. Four polysaccharide-iron (III) complexes (0–10 mg/mL, 0.1 mL) were mixed with ABTS reagent (3.9 mL), and the absorbance was measured at 734 nm after the reaction for 10 min, protected from light. The ABTS radical scavenging activity was measured using Equation (3), and the IC_50_ was calculated. Equal concentrations of SCCP were prepared and measured in the same manner; the same concentration of ascorbic acid served as a positive control.

(3) $$\mathrm{ABTS}\;\mathrm{radical}\;\mathrm{scavenging}\;\mathrm{activity}(\%)=(1-\frac{A_{1}-A_{2}}{A_{0}})\times 100$$

where *A*_0_ is the absorbance without the sample, *A*_1_ is the absorbance of the SCCP-Fe complex, and *A*_2_ is the absorbance without the ABTS+ reagent.

### 3.6. Determination of Hypoglycemic Activity In Vitro

#### 3.6.1. α-Glucosidase Inhibitory Activity

Four polysaccharide-iron (III) complexes (0–10 mg/mL, 1 mL) were mixed with α-glucosidase (1 U/mL, 0.1 mL) and a PBS buffer solution (pH = 6.8, 2 mL) and reacted at 37 °C, and p-nitrophenyl-β-d-galactopyranoside (10 mmol/L, 0.25 mL) and glutathione solution (3 mmol/L, 0.1 mL) were added. The reaction was carried out in a water bath at 37 °C. The reaction was terminated by adding sodium carbonate solution (0.1 mol/L, 2 mL) for 10 min, and the absorbance was measured at 400 nm. The inhibition rate was calculated using Equation (4), as shown below. Simultaneously, the concentration of polysaccharide-iron (III) complexes at 50% enzyme inhibition was calculated. Equal concentrations of SCCP was prepared and measured in the same manner, and the same concentration of acarbose served as a positive control [55].

(4) $$\mathrm{Inhibition}\;\mathrm{rate}(\%)=\frac{\left(A_{0}-A_{2})-(A_{1}-A_{3}\right)}{\left(A_{0}-A_{2}\right)}\times 100$$

where *A*_0_ is the absorbance without the sample, *A*_1_ is the absorbance of the sample, *A*_2_ is the absorbance of PBS alone, and *A*_3_ is the absorbance of the sample without α-glucosidase.

#### 3.6.2. α-Amylase Inhibitory Activity

Four polysaccharide-iron (III) complexes (0–10 mg/mL, 1 mL) were mixed with a soluble starch solution (0.01 g/mL, 0.3 mL) and α-amylase solution (2 U/mL, 0.3 mL) for 10 min at 37 °C. Next, the 3,5-dinitro salicylic acid solution (0.5 mL) was added, heated in a boiling water bath for 5 min, fixed to the same volume, and the absorbance was measured at 540 nm. The inhibition rate was calculated using Equation (5), and the concentration of polysaccharide-iron (III) complexes at 50% enzyme inhibition was determined. Acarbose was used as a positive control [56]:

(5) $$\mathrm{Inhibition}\;\mathrm{rate}\;(\%)=\frac{\left(A_{0}-A_{2})-(A_{1}-A_{3}\right)}{\left(A_{0}-A_{2}\right)}\times 100$$

where *A*_0_ is the absorbance without the sample, *A*_1_ is the absorbance of the sample, *A*_2_ is the absorbance without the sample and esorblase solution, and *A*_3_ is the absorbance without α-amylase.

### 3.7. Statistical Analysis

All the experiments were independently repeated at least thrice. A data analysis was performed using analysis of variance (ANOVA) and Tukey’s test for multiple comparisons to determine the significance of the differences. The results are presented as mean ± standard deviation (SD). The statistical analyses were performed using SPSS 18.0 (SPSS, Chicago, IL, USA). All the figures were generated using Origin 2018 software (OriginLab Corp., Northampton, MA, USA).

## 4. Conclusions

In this study, we prepared and characterized four sweet corn cob polysaccharide-iron (III) complexes at different temperatures (40 °C, 50 °C, 60 °C, and 70 °C) using a co-thermal synthesis of ferric trichloride. The four complexes’ in vitro simulated release, antioxidant, and hypoglycemic abilities were assessed. This indicated that iron (III) could successfully bind to polysaccharides to form polysaccharide-iron (III) complexes at all four temperatures. The binding mode is most likely formed by polysaccharide-forming β-FeOOH iron nuclei with −OH and −OOH. We also found that the introduction of iron (III) barely changed the basic structure of the polysaccharide, and it retained an amorphous crystal structure. Moreover, the thermal stability of the complexes significantly increased. We found the mass loss rates at 30–600 °C were 48.75%, 47.15%, 45.07%, and 48.76% in four complexes, respectively, which were remarkably lower than those of SCCP (81.82%). Next, we determined the in vitro release of these four complexes. The total iron (III) release rates of the complexes in vitro were 90.27 ± 1.23%, 92.73 ± 1.47%, 92.05 ± 2.48%, and 91.62 ± 1.46%, respectively. This suggested that the polysaccharide-iron (III) complex was rapidly released and equilibrated in vitro. Finally, we measured the in vitro activity of these complexes. This indicates that all four polysaccharide-iron (III) complexes showed significantly higher in vitro antioxidant activity and inhibition of α-glucosidase and α-amylase than SCCP. Furthermore, the highest levels of biological activity were observed in SCCP-Fe-B (50 °C) and SCCP-Fe-C (60 °C). We presume this is also related to the stable β-FeOOH iron nuclei formed in the polysaccharides by sufficient iron (III), which facilitates their biological activity. We discovered that the polysaccharides derived from sweet corn cobs, a plant agricultural byproduct, could be used as a potential iron (III) supplement to prepare polysaccharide-iron (III) complexes with high bioactivity. We will continue to investigate the mechanism of sweet corn cob polysaccharide-iron (III) complexes in alleviating oxidative stress in diabetic animal models and provide theoretical references for future natural product iron supplements.

## Figures and Tables

**Table 1:** Chemical and monosaccharide compositions of polysaccharides.

Samples	SCCP	SCCP-Fe-A	SCCP-Fe-B	SCCP-Fe-C	SCCP-Fe-D
Total carbohydrates (%)	72.91 ± 0.19	74.21 ± 2.37 *	72.52 ± 4.29	73.36 ± 3.08	71.27 ± 2.85
Uronic acid (%)	9.58 ± 0.12	4.21 ± 0.35 **	3.16 ± 0.26 **	2.01 ± 0.13 **	2.96 ± 0.34 **
Protein (%)	1.16 ± 0.05	1.26 ± 0.17	1.04 ± 0.22	0.97 ± 0.14	1.07 ± 0.19
Fe (%)	0.07 ± 0.01	12.75 ± 1.26 **	15.67 ± 1.48 **	21.09 ± 2.29 **	14.82 ± 1.31 **
pH	6.85 ± 0.13	6.91 ± 0.12	6.92 ± 0.23	6.92 ± 0.18	6.90 ± 0.15
Monosaccharide compositions (mol %)					
Man	4.613	1.504	2.437	2.946	2.770
Glc	66.609	65.822	63.368	62.482	61.644
Gal	10.135	12.890	12.561	12.770	12.433
Ara	7.157	13.391	13.523	12.548	12.857
Fuc	5.878	1.200	2.438	2.682	3.543

*: *p* < 0.05 compared to the control group (SCCP); **: *p* < 0.01 compared to the control group (SCCP).

## Data Availability

The data presented in this paper are available on request from the first author and corresponding author.

## Sample Availability

Samples of the compounds of this study are available from the corresponding author upon reasonable request.
